# Efficacy study of juxta-anastomotic stenosis of the arteriovenous fistula via percutaneous transluminal angioplasty with different accesses to the arteries and veins

**DOI:** 10.3389/fmed.2026.1883032

**Published:** 2026-07-22

**Authors:** Zhongyuan Lv, Jiemin Yang, Yang Xu, Yuhong Chen, Liuwei Zhao, Peiyu Wang, Xiaomin Dong, Yang Qin, Xinyue Wang, Qingqing Xiao, Jingsong Mao

**Affiliations:** 1 Department of Interventional Vascular Surgery, The Affiliated Qingyuan Hospital (Qingyuan People’s Hospital), Guangzhou Medical University, Qingyuan, China; 2 Department of Vascular Intervention, Affiliated Hospital of Guilin Medical University, Guilin, Guangxi, China; 3 Guilin Medical University, Guilin, Guangxi, China; 4 Ward of Cardiothoracic and Vascular Surgery, Liuzhou Traditional Chinese Medical Hospital, The Third Affiliated Hospital of Guangxi University of Chinese Medicine, Liuzhou, China; 5 Department of Pharmacy, Affiliated Hospital of Guilin Medical University, Guilin, Guangxi, China; 6 Department of Graduation, Affiliated Hospital of Guilin Medical University, Guilin, Guangxi, China

**Keywords:** access approach, arteriovenous fistulas, juxta-anastomotic stenosis, patency, percutaneous transluminal angioplasty

## Abstract

**Objective:**

Juxta-anastomotic stenosis is a leading cause of dysfunction in arteriovenous fistulas (AVFs) for hemodialysis. Percutaneous transluminal angioplasty (PTA) is the primary treatment, but the optimal access approach remains unclear. This study compared the efficacy of arterial versus venous access PTA for treating juxta-anastomotic AVF stenosis and identified risk factors for post-PTA patency loss.

**Methods:**

This retrospective study analyzed 267 patients who underwent PTA for AVF stenosis. Patients were divided into vein and artery groups based on access route. The study compared primary patency rates, intervention interval times, restenosis rates, and postoperative complications between the two groups. Factors affecting AVF patency were analyzed by Cox regression analysis and Kaplan–Meier curves. Subgroup analysis was used to evaluate the variations in the relationships between relevant variables and patency under different access route baselines.

**Results:**

Compared with venous access, arterial access PTA demonstrated superior primary patency, with 6-month rates of 76.6% vs. 46.0% (*p* < 0.001). The arterial group had lower restenosis (23.4% vs. 54.0%, p < 0.001). The incidence of postoperative complications was lower in the arterial group than in the vein group (18.8% < 47.8%, *p* < 0.05). Diabetes, fibrin degradation products (FDPs) and D-Dimer independently predict patency loss. This study revealed for the first time that FDP levels have a U-shaped relationship with AVF patency (*p* = 0.009). Diabetes was a stronger predictor in the arterial group, whereas FDP and D-Dimer were more important in the vein group (*p* < 0.05).

**Conclusion:**

Compared with venous access, arterial access PTA offered superior primary patency and lower restenosis for juxta-anastomotic AVF lesions. Diabetes, elevated FDP, and D-Dimer predicted patency loss, with the prognostic impact varying according to the vascular access route.

## 1 Introduction

End-stage renal disease (ESRD) poses a significant global health burden, with an estimated prevalence of 10.8% in China and an increasing incidence as populations age. Hemodialysis remains the predominant treatment modality for ESRD, and maintaining functional vascular access is crucial for its success (1–3). Compared with grafts or catheters, arteriovenous fistulas (AVFs) are the preferred access type, offering superior long-term patency and fewer complications (3, 4). However, peri-anastomotic stenosis of AVF, which usually occurs within 5 cm of the arteriovenous anastomosis, and secondary thrombosis are the main causes of vascular access dysfunction. Thus, AVF dysfunction is a major cause of increased hospitalization in hemodialysis patients (1, 5–8).

The pathogenesis of AVF stenosis is multifactorial, primarily driven by venous neointimal hyperplasia (VNH) and inadequate outward remodeling (9, 10). Hemodynamic alterations, surgical stressors, and inflammatory stimuli can all contribute to the production of proinflammatory factors that promote inadequate outward remodeling, resulting in reduced AVF blood flow, neointimal hyperplasia, luminal stenosis, and thrombosis (5). Neoendometrial lesions with AVF stenosis are primarily composed of smooth muscle cells (SMCs) and many macrophages (11, 12). When inflammation causes endothelial injury, infiltrating macrophages express a variety of cytokines that initiate the proliferation and migration of SMCs, resulting in intimal hyperplasia (13, 14).

Percutaneous transluminal angioplasty (PTA) has emerged as the first-line therapy for treating AVF stenosis, with technical success rates ranging from 90–95% for juxta-anastomotic lesions (14–16). By restoring luminal patency, PTA prolongs the AVF lifespan and reduces the need for surgical revision (17). However, post-PTA restenosis remains a challenge, with 1-year primary patency ranging from 40–60% (14, 16, 18). Studies have shown that PTA treatment via narrowed AVF arterial access and venous access is a feasible treatment method characterized by safety and feasibility. Traditionally, transvenous side channel PTA is the first choice for the treatment of AVF narrow access. However, in recent years, arterial access PTA has been widely used, but its comparative efficacy and risk factors for post-PTA patency loss have not been well elucidated. The difference in the effectiveness of PTA treatment under different paths has not been fully studied. This study aimed to compare the efficacy of arterial versus venous access PTA for juxta-anastomotic AVF stenosis and identify predictors of post-PTA patency loss. These findings may inform individualized PTA strategies and guide future research to optimize AVF interventions and improve patient outcomes.

## 2 Methods

### 2.1 Patients and procedures

**Figure 1:**
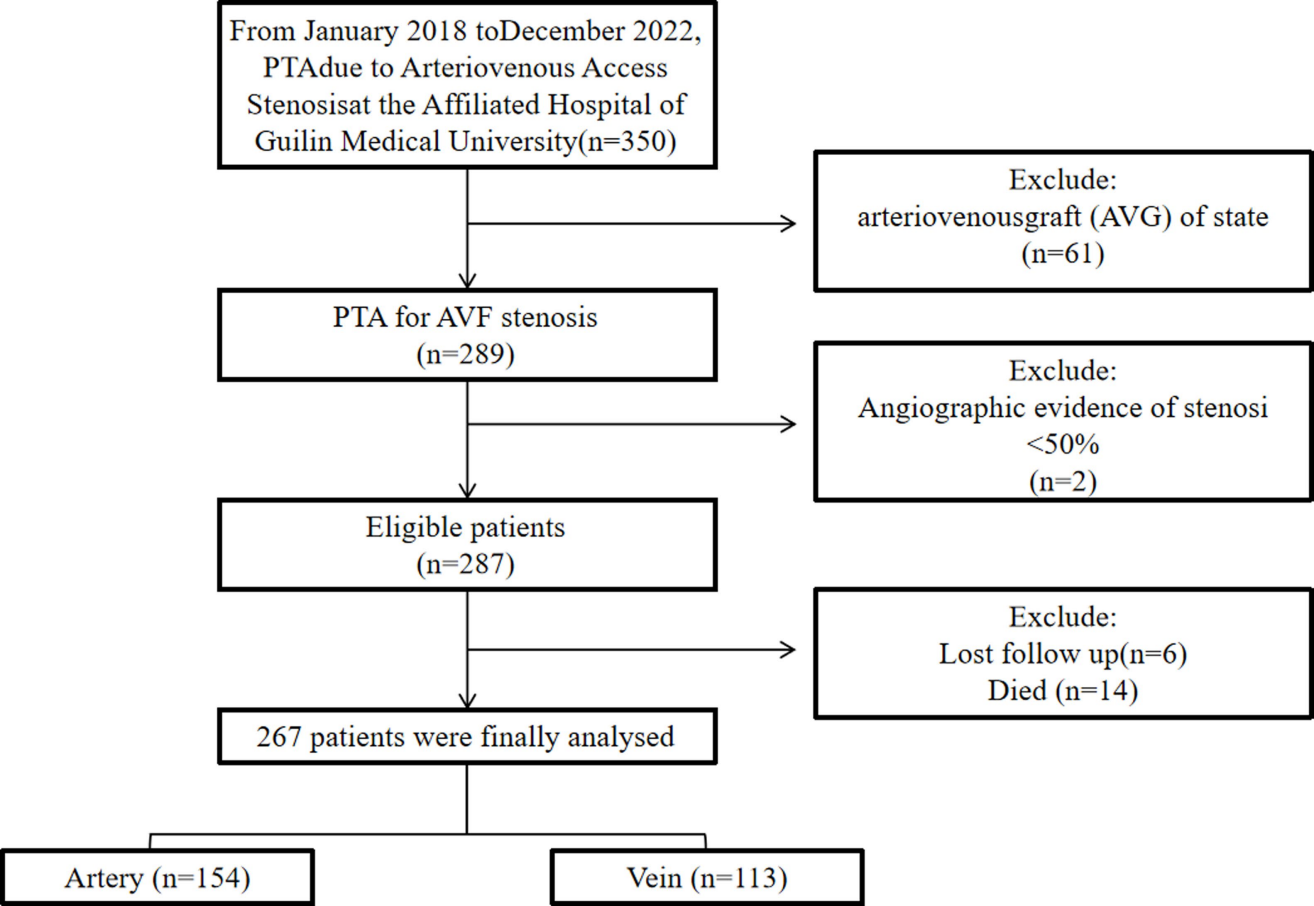
Flow diagram of the study population.

This retrospective single-center study included 350 patients who underwent PTA for juxta-anastomotic AVF stenosis at the Affiliated Hospital of Guilin Medical University institution between January 2018 and December 2022. Juxta-anastomotic stenosis is defined as a stenosis lesion within 5 cm of the arteriovenous anastomosis (19, 20). The 350 patients were screened according to the following inclusion and exclusion criteria. Inclusion criteria: (1) Informed consent has been obtained. (2) Male or female ≥21 years of age. (3) Established and used for hemodialysis arteriovenous fistula access. (4) Angiography or vascular color Doppler ultrasound showing peri-anastomotic stenosis of the arteriovenous endovascular fistula or with the following conditions: insufficient blood flow on dialysis, high venous pressure, and difficulty in puncturing during dialysis. (5) Juxta-anastomotic stenosis: the distance between the stenotic lesion and the anastomotic site is ≤5 cm. Exclusion criteria: (1) AVG internal fistula dialysis. (2) Lower limbs hemodialysis pathway. (3) <50% stenosis near the anastomotic site of autogenous arteriovenous fistula indicated by angiography or vascular color Doppler ultrasound. (4) Patients who were lost to follow-up or needed re-intervention for other reasons unrelated to the target lesion during the follow-up period, including patients who underwent kidney transplantation and patients who died, were excluded. The flow chart of the enrollment population is shown in Figure 1. Patients were divided into the vein group and the artery group based on the use of arterial or venous access for PTA of juxta-anastomotic AVF stenosis.

The Institutional Review Committee of the Affiliated Hospital of Guilin Medical University approved the retrospective analysis of the patients’ clinical data (ethics approval number: 2023YJSLL-40).

### 2.2 PTA procedure

Intra-arterial and intravenous percutaneous transluminal angioplasty included routine disinfection with a sterile surgical towel, local infiltration with 2% lidocaine injection, and a modified Seldinger method to successfully puncture the radial artery (cephalic vein/brachial vein) at the level of the elbow joint. The guide wire was confirmed to enter the true lumen of the blood vessel, and the 6FR vascular sheath was placed under the guidance of the guide wire. Intravascular heparin (3,000 IU) was used to prevent intravascular thrombosis. The guide wire and the angiographic catheter were inserted into the AVF, and the angiographic agent was properly injected at the beginning of the occlusion, within the occluded segment, and near the cardiac side of the segment. The degree of stenosis was further clarified. A balloon dilatation catheter (with a diameter range of 3–7 mm) was then used to predilute the stenotic vessels. The balloon was gradually inflated with a balloon dilatation pump for 60 s, and the procedure was repeated 2–3 times until the stenotic segment was dilated again. At the end of surgery, bandaging and fixation were performed.

### 2.3 Data collection and definition

Data was systematically collected from electronic medical records of eligible patients over a defined study period. Variables included anatomical factors, clinical characteristics, and laboratory parameters were collected. All blood samples were collected from fasting venous blood in the morning (before narrowing to PTA occurs).

Complications included thrombus, occlusion, pseudoaneurysm and aneurysmal.

Technical success was defined as residual stenosis of less than 30%. Clinical success was indicated by a palpable thrill, audible bruit, and successful hemodialysis.

### 2.4 Study endpoints and follow up

All patients were followed up with color Doppler ultrasound and effective hemodialysis at 3, 6 and 12 months after the operation.

The primary endpoint was PTA to AVF restenosis. Restenosis rate: probability of restenosis 6 months after PTA (<50% diameter reduction).

### 2.5 Statistical methods

Continuous variables were presented as mean ± standard deviation (SD) or median (interquartile range: 25th to 75th percentile) and were compared using either t-tests or Wilcoxon rank-sum tests, depending on the data distribution. Categorical variables are presented as percentages and were analyzed with chi-square tests. Multivariable COX regression was performed to identify parameters associated with primary patency at 6-month at follow-up, variables with *p* < 0.2 on univariable analysis were included in the multivariable model. The differences in primary patency among populations with different thresholds of risk factors were plotted using Kaplan–Meier (K-M) curves, and the differences were analyzed using the log-rank test. In addition, Restricted Cubic Spline (RCS) regression curves were used to evaluate linearity, examine dose–response relationships between relevant variables and patency, and determine the threshold value.

Subgroups were assessed in different ways, the potential changes in the relevant relationships between variables and flow, the heterogeneity between the subgroups was evaluated with multivariate Cox regression, and the interactions between subgroups and unobstructed relationships were evaluated with a likelihood ratio test.

Factors with *p* < 0.05 were considered statistically significant. Through these methods, the purpose of this study is to analyze the long-term patency of AVF stenosis affected by PTA in different ways. Statistical analysis was performed via SPSS Statistics v.22 (IBM Corporation, Armonk, NY, USAR), R4.3.1 (R Core Team 2023), and GraphPad Prism 9.5.

## 3 Results

### 3.1 Baseline characteristics of the study population

Of the 350 patients who met the initial inclusion criteria, 267 were ultimately selected for further analysis after applying the exclusion criteria: 154 in the arterial group and 113 in the venous group. Table 1 shows the baseline characteristics of all patients, including a total of 140 males and 127 females, with a mean age of 59 years. In this study, the baseline levels of patients in the arterial and venous groups were compared (Table 2), and the differences were not statistically significant (*p* > 0.05).

**Table 1:** Univariate and multivariate Cox regression analysis affecting 6 months post-PTA.

Predictors	Univariable analysis		Multivariable analysis	
	HR (95% CI)	*p*-value	HR (95% CI)	*p*-value
Sex	0.75 (0.5, 1.12)	0.155	0.77 (0.51, 1.18)	0.232
Age	1.01 (0.99, 1.02)	0.499		
BMI	1.01 (0.96, 1.07)	0.767		
Hypertension	0.93 (0.56, 1.53)	0.767		
Diabetes	1.70(1.15, 2.53)	0.008	1.67 (1.11, 2.50)	0.013
Carcinoma	1.50 (0.66, 3.42)	0.364		
Peripheral vascular disease	0.91 (0.61, 1.36)	0.648		
Hyperlipidemia	0.90 (0.40, 2.06)	0.808		
Surgical approach (Artery/Vein)	0.37 (0.25, 0.56)	<0.001	0.36 (0.24, 0.55)	<0.001
Preoperative blood flow	0.99 (0.99, 1.00)	0.045	1.00 (1.00, 1.00)	0.092
WBC	0.98 (0.91, 1.06)	0.616		
Neutrophil ratio	0.28 (0.04, 1.8)	0.182	0.17 (0.02, 1.14)	0.068
FDP	1.06 (1.01, 1.12)	0.033	1.07 (1.02, 1.15)	0.01
D-Dimer	1.08 (1.03, 1.14)	0.012	1.08 (1.02, 1.14)	0.005
Creatinine	0.99 (0.99, 1.00)	0.394		

BMI, body mass index; WBC, white blood cell; FDP, Fibrin degradation products.

**Table 2:** Baseline patient demographics and medical history.

Variable	Overall (*n* = 267)	Vein (*n* = 113)	Artery (*n* = 154)	*p-*value
Sex				0.326
Male	127 (47.6)	57 (51.3)	69 (44.8)	
Female	140 (52.4)	55 (48.7)	85 (55.2)	
BMI	22.2 (20.2, 24.3)	22.1 (19.9, 24.2)	22.6 (20.6, 24.3)	0.159
Age (y)	59.0 (50.0, 68.0)	57.0 (48.0, 67.0)	60.0 (51.0, 68.0)	0.325
Hypertension	219 (82.0)	96 (85)	123 (79.9)	0.285
Diabetes	112 (41.9)	44 (38.9)	68 (44.2)	0.393
Carcinoma	13 (4.9)	3 (2.7)	10 (6.5)	0.15
Peripheral vascular disease	113 (42.3)	51 (45.1)	62 (40.3)	0.426
Hyperlipidemia	18 (6.7)	5 (4.4)	13 (8.4)	0.196
Preoperative blood flow	139.0 (93.0, 200.0)	127.0 (75.0, 194.0)	150.0 (97.5, 200.0)	0.128
WBC	6.7 (5.3, 8.2)	6.7 (5.4, 8.2)	6.7 (5.3, 8.2)	0.612
Neutrophil ratio	0.7 (0.6, 0.8)	0.7 (0.6, 0.8)	0.7 (0.6, 0.8)	0.724
FDP	2.9 (1.6, 5.2)	2.5 (1.5, 5.2)	3.2 (1.7, 5.1)	0.157
D-Dimer	0.7 (0.4, 1.4)	0.6 (0.3, 1.3)	0.8 (0.4, 1.6)	0.081
Creatinine	865.0(635.5, 1105.0)	817.8(569.0, 1052.0)	891.1(660.1, 1106.5)	0.198

Continuous parameter usually presented as means± SD. BMI, body mass index.

### 3.2 The study endpoints

**Figure 2:**
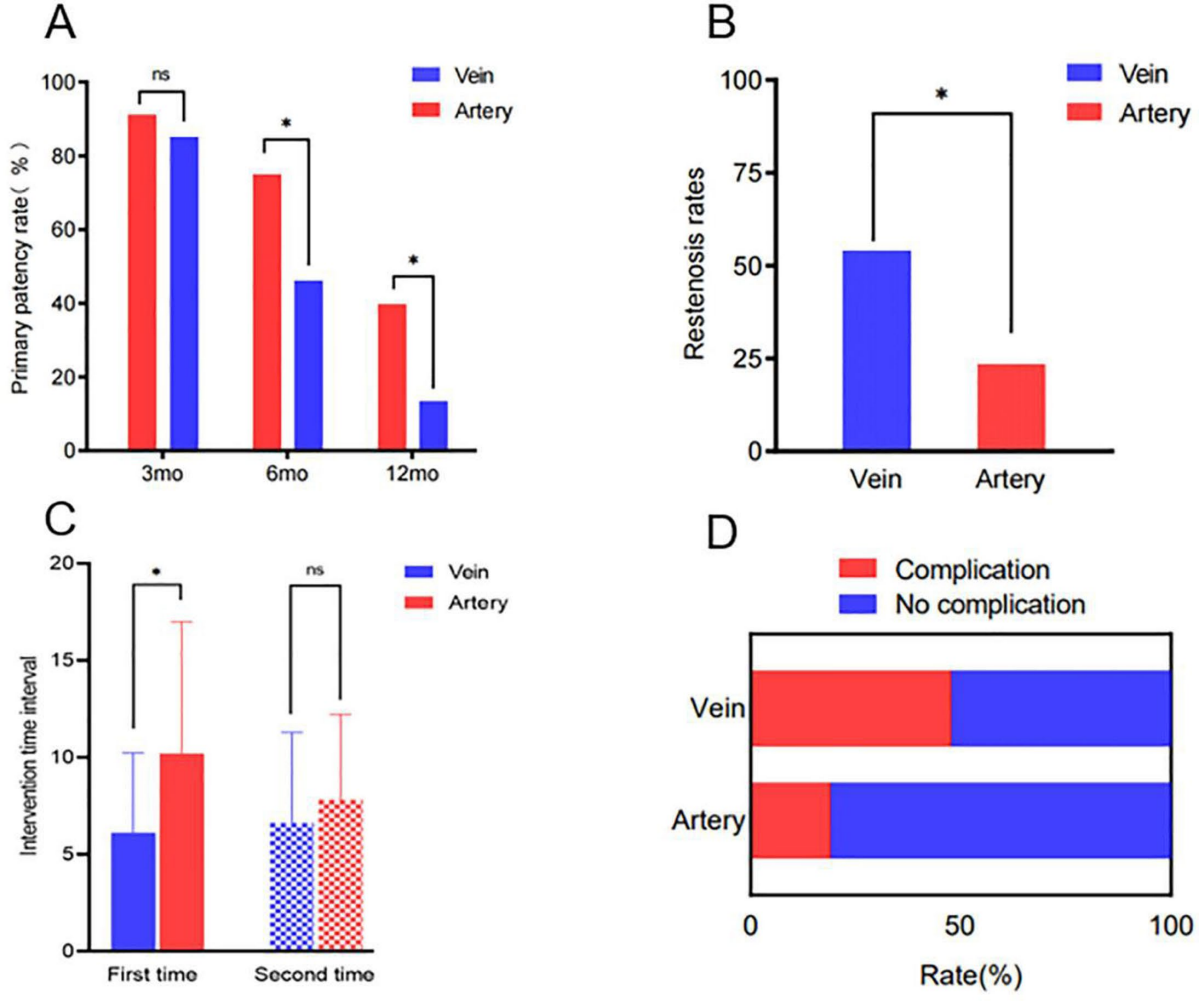
A comparison of the primary patency rate, restenosis rate and intervention time interval revealed that the arterial group was superior to the vein group. A higher primary patency rate **(A)**, a lower restenosis rate **(B)** and a longer intervention time interval **(C)** were observed at 6 months. **(D)** Comparison of complications between the venous and arterial groups.

The success rate of tube placement was 100%, the technical success rate was 100% in both groups in the present study, with clinical success rates of 98.7 and 92.2% (*p* > 0.05). Among the 267 patients with perianastomotic stenosis, the primary patency rates at 3, 6 and 12 months after surgery were 90.6, 63.4 and 28.3% (*p* < 0.05). At 3 months postoperatively, the patency rates of the arterial and vein groups were 92.9 and 88.5% (p > 0.05); at 6 months postoperatively, the patency rates were higher in the arterial group than in the vein group (76.6% > 46%, p < 0.05), suggesting that the arterial end paths had better Short- and long-term patency after PTA treatment (Figure 2A).

### 3.3 Complication

The main reasons for decreased patency after PTA are restenosis and endovascular thrombosis, and in this study, it was found that stenosis sites required an average of 2.72 PTA treatments to maintain patency. 36 patients in the arterial group and 61 patients in the vein group had restenosis requiring another PTA during the 6-month postoperative follow-up period, and the restenosis rate was lower in the arterial group than in the vein group (23.4% < 54%, *p* < 0.05) (Figure 2B). The mean time between interventions was better in the arterial group than in the vein group (10.1 months >6.6 months, *p* < 0.05) (Figure 2C). Post-operative complications occurred in 83 of 267 patients during follow-up; the incidence of postoperative complications was lower in the arterial group than in the vein group (18.8% < 47.8%, *p* < 0.05) (Figure 2D). Specific complications include endovascular thrombosis, occlusion, pseudoaneurysm, aneurysmal dilatation (Table S3).

Univariate Cox regression analysis revealed that diabetes, preoperative blood flow, approach, FDP and D-Dimer were significantly related to the patency of the PTA at 6 months after the operation. Multivariate Cox regression analysis revealed that diabetes, approach, FDP, and D-Dimer were predictors of lesion patency (Table 1).

**Figure 3:**
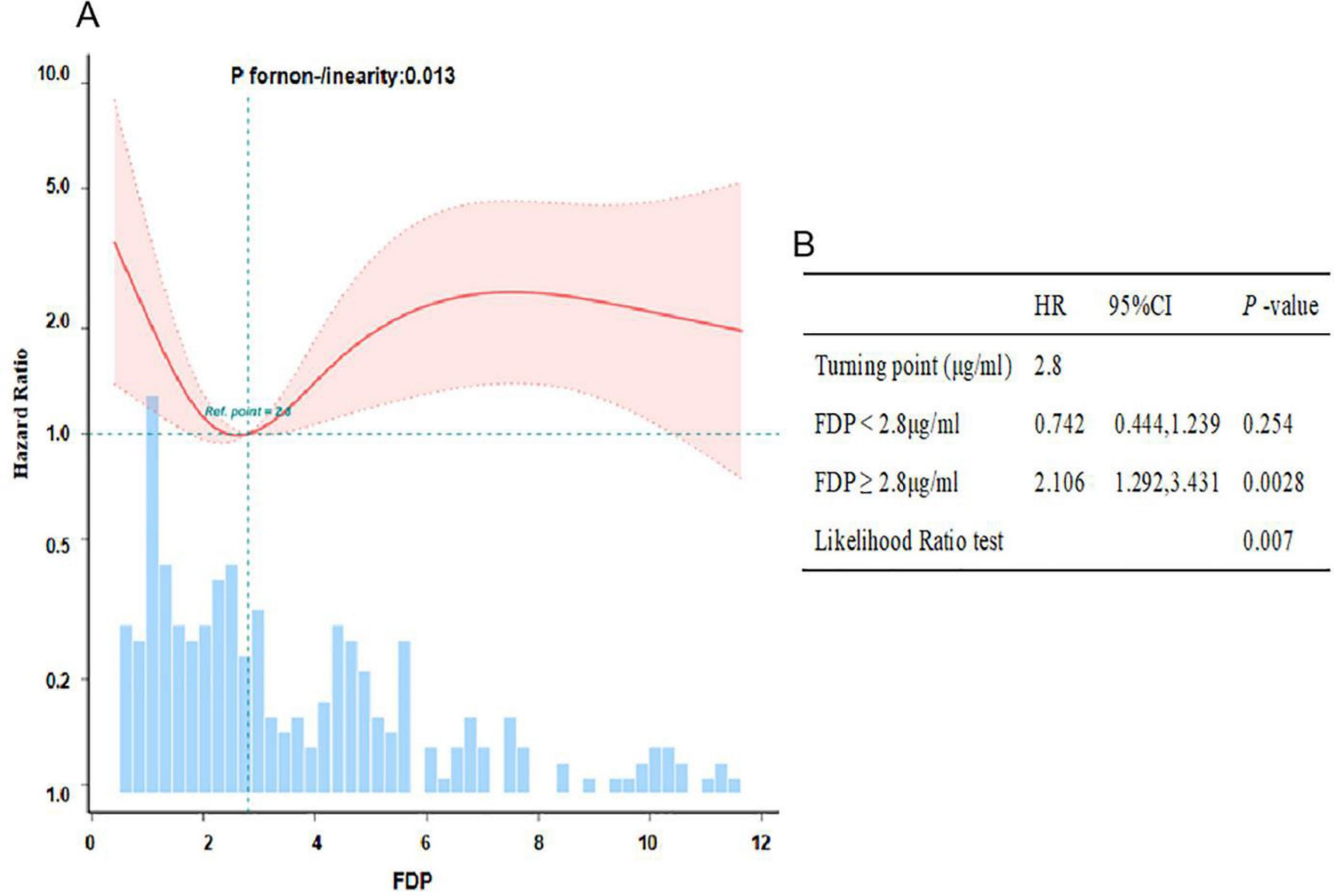
The nonlinear relationship between FDP and the fluency rate in the AVF **(A)**. Threshold effect analysis of FDP and the patency rate of AVF via Cox regression models **(B)**. Cox regression models were adjusted for all covariates (the upper limit of 98%).

The RCS regression curve revealed a U-shaped relationship between FDP and patency (*p* = 0.013) (Figure 3A). The inflection point was found to be approximately 2.8 μg/mL, and to the right of the inflection point, the hazard ratio (HR) was 2.106 (95% CI: 1.292–3.431, *p* = 0.0028). In the threshold analysis, the *p* value of the likelihood ratio test was less than 0.007 (Figure 3B). The results revealed that the risk of restenosis was lowest when the FDP was 2.8 μg/mL, and the patency was better. The risk of restenosis increased when the FDP was greater than 2.8 μg/mL.

The Cox regression analysis revealed that diabetes was a risk factor for postoperative PTA patency in the arterial group (Supplementary Table S1), whereas D-Dimer and FDP were risk factors for postoperative PTA patency in the vein group (Supplementary Table S2).

### 3.4 Subgroup analysis

**Figure 4:**
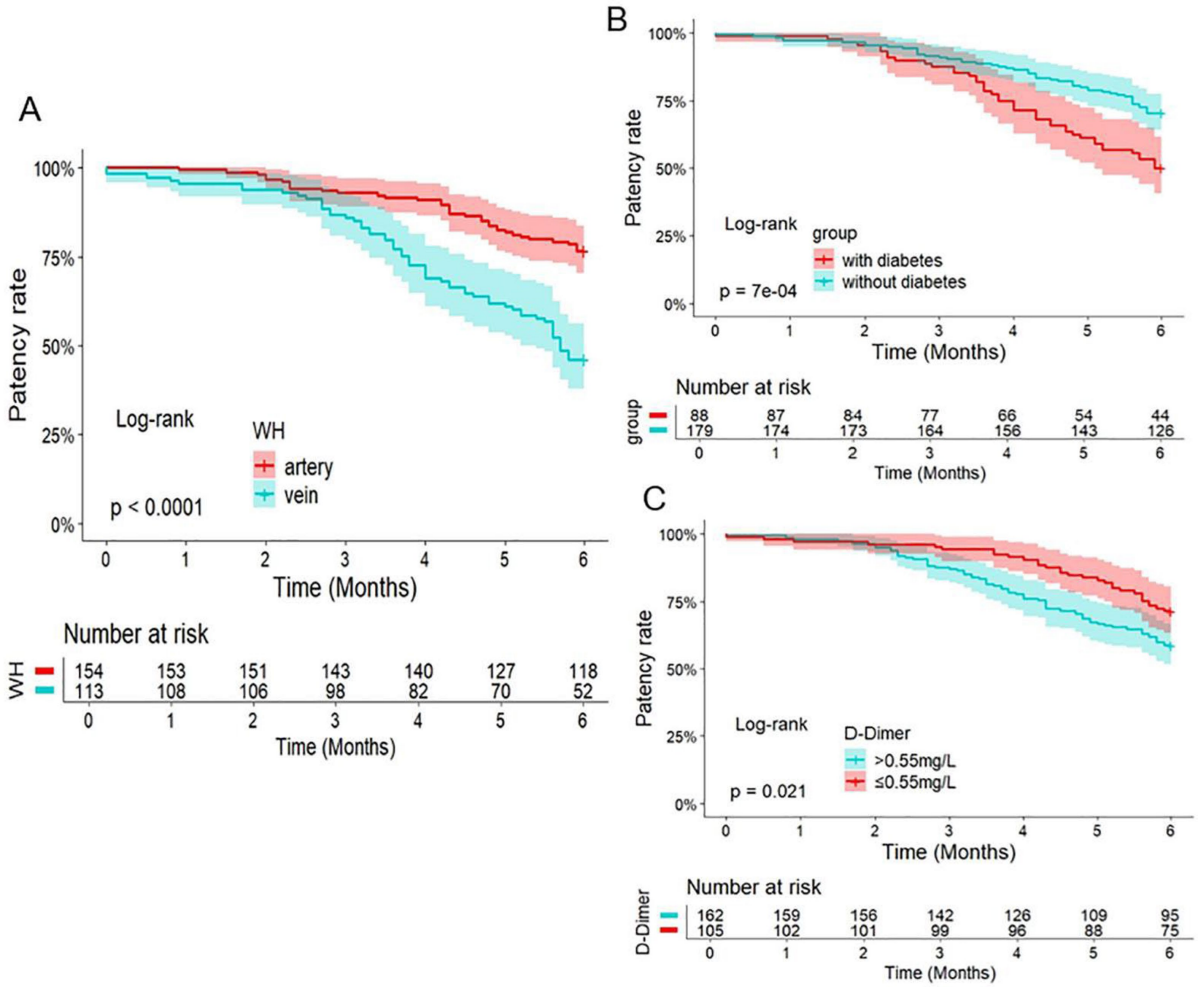
Kaplan–Meier curve was used to verify the risk factors and protective factors that affect the rate of AVF stenosis after interventional treatment, and the related factors included access **(A)**, diabetes **(B)**, and D-Dimer **(C)**.

To verify the risk factors and protective factors affecting patency, a subgroup analysis was performed for diabetes, approach and D-Dimer (Figure 4). Based on the commonly used clinical cut-off value of 0.55 mg/L for D-Dimer. The Kaplan–Meier curve revealed that the patency rate was good in patients with the artery access (Figure 4A), without diabetes patients (Figure 4B) and D-Dimer ≤0.55 mg/L (Figure 4C) after PTA (*p* < 0.05). Therefore, diabetes and D-Dimer >0.55 mg/L were risk factors for patency, and the patency of PTA via arterial access was better than that via venous access.

To further verify the risk factors and protective factors that affect the patency rate of arterial and venous lesions, a grouping analysis of diabetes, FDP and D-Dimer was performed (Supplementary Figure S1). Without diabetes patients had good patency in the arterial group (*p* < 0.05) (Supplementary Figure S1A). Patients with D-Dimer ≤0.55 mg/L and FDP < 2.8 μg/mL had better short-term primary patency rates in the vein group (Supplementary Figures S1B,C).

There were significant differences in the risk factors for PTA among the arterial, venous and general populations. These RCS regression studies demonstrated the advantage of patency in FDP subgroups after the arterial approach.

**Figure 5:**
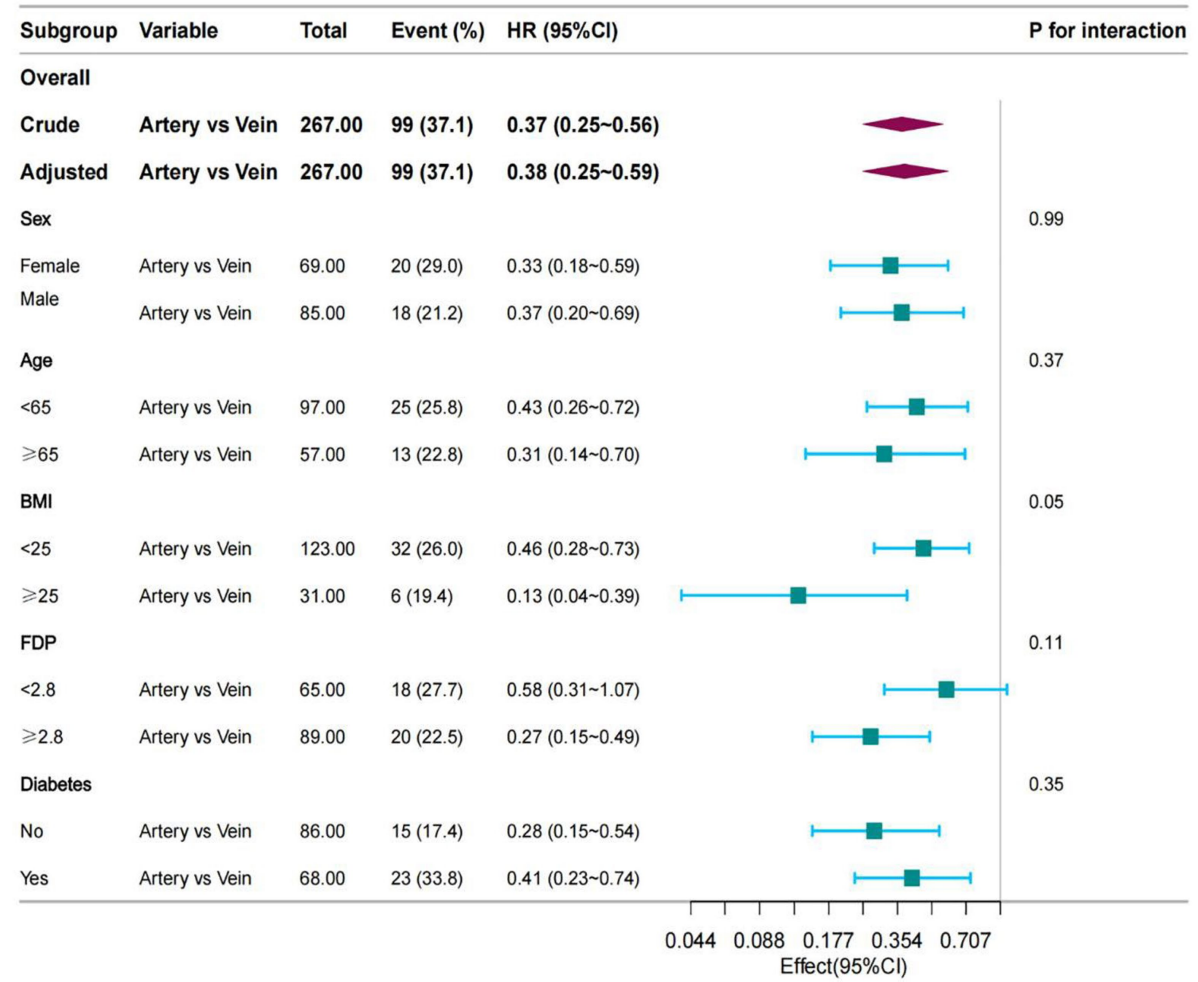
The graph is drawn from the different subgroups: sex, age (20–50 years vs. >50 years), diabetes status, FDP (<2.8 μg/mL vs. ≥2.8 μg/mL), and body mass index (<25 kg/m^2^ vs. ≥25 kg/m^2^) to analyze the relationship between access and patency.

Stratified analyses of laboratory subgroups were performed to assess the potential impact of the relationship between relevant variables and patency under different access modalities as benchmarks (Figure 5). The results of the analysis in the total population overall, HR = 0.37, demonstrated a 63% reduction in the risk of restenosis with arterial versus venous access, suggesting that the difference between arterial PTA for AVF stenosis versus venous PTA for AVF stenosis in the overall population was statistically significant. After stratification by BMI, sex, age, diabetes mellitus, and FDP, the hazard ratio (HR) was less than 1, all to the left of the null line, and arterial access PTA treatment was analyzed with a *p*-value greater than 0.05, thus, the difference between subgroups was not significant in terms of strata, further confirming the arterial access stability of the patency model after PTA treatment.

## 4 Discussion

AVF is the key to maintaining quality of life among hemodialysis patients. AVF maturation is a complex, long-term process during which arteriovenous anastomosis progressively increases the inner diameter of arteries and veins, increases blood flow and shear forces, stimulates endothelial-dependent vasodilation responses of arteries and veins, thereby dilating inflow into and out of arteries and promoting venous arterialization, which are mainly mediated by the activation of nitric oxide (NO) and matrix metalloproteinases (MMPs) (13, 20, 21). However, the maturation of AVFs takes longer, and as the duration of dialysis increases, vascular calcification caused by chronic diseases such as diabetes and hypertension also affect AVF maturation. The main cause of AVF maturation failure is stenosis and thrombosis caused by neointimal hyperplasia surrounding the anastomosis (9, 13, 15).

The effectiveness of the PTA has been confirmed in many studies, but owing to elastic recovery and the recurrence of intimal hyperplasia, the PTA may be linked to an increased incidence of restenosis, and the unobstructed relationship is still very poor (22, 23). AVF has a greater than 50% probability of restenosis within a few months and requires multiple repetitions of PTA therapy to maintain hemodynamic stability (24). In a study by Yang et al. (25) approximately 70–86% of patients required two to three interventions in the first year after the formation of internal fistulae to maintain function. In the present study, the stenosis sites required an average of 2.72 PTA treatments to maintain access (26).

Currently, PTA entry methods include the venous approach and the arterial approach. Studies have demonstrated that arterial access is a safe and effective method, with reported complication rates ranging from 1.3 to 3.4% (27, 28). Transracial arterial access provides better exposure of the peri-anastomotic area, allowing easier lesion assessment and guidewire passage and avoiding damage to the vessel wall from repeated venous punctures, and the rich anastomotic and collateral network between the ulnar and radial arteries ensures blood supply to the fingers (29–31). The advantages of arterial route may become the standard choice in the future, especially for certain high-risk patient groups, such as diabetic patients. Although venous access PTA has traditionally been the modality of choice for the treatment of AVF stenosis, its advantages include ease of maneuvering, rapid hemostasis, early bed mobility and fewer complications (32, 33). However, retrograde intravenous injection of contrast agent can result in poor visualization of the veins and arteries adjacent to the anastomosis of the stenotic lesion, and in AVFs with venous stenosis combined with proximal stenosis, venous access PTA treatment does not simultaneously resolve the stenotic lesion. And the passage of the guidewire and balloon catheter may be limited if the angle of the endovascular anastomosis is acute (34, 35).

In the present study, arterial access PTA for AVF stenosis demonstrated a higher rate of long-term patency, a lower rate of restenosis, longer intervals between interventions, and a lower incidence of complications than venous access treatment did. Further validation of the risk factors affecting the patency associated with after PTA for AVF stenosis revealed that diabetes, FDP, and D-Dimer were risk factors affecting the patency associated with PTA for AVF stenosis.

Maturation and stenosis of arteriovenous fistulas (AVFs) are both closely related to hemodynamic changes. Decreased endovascular blood flow during hemodialysis may predict AVF malfunction (36). In diabetic patients, the production of vascular endothelial cells is reduced, the expression of nitric oxide synthase (NOS) is decreased, and the high glucose state inhibits the expression of MMP-2 and MMP-9 in endothelial and smooth muscle cells, attenuates the response to shear forces, and reduces the arteriolar dilation response (37). In cellular studies, diabetes impairs the proliferation and migration of smooth muscle cells and reduces the expression of VEGF in ischemic lesion tissue (38), which is likely one of the main reasons for the influence of AVF patency.

FDP and D-Dimer are involved in the development of thrombophilia. Thrombus deposition within the vein wall and on its surface is a response to the risk of restenosis, which is triggered by vascular lesions and PTA treatment-induced vascular endothelial cell injury (39). FDP promotes the proliferation of vascular endothelial cells, SMCs, and fibroblasts, which leads to hardening and stenosis of the vessel wall (40).

We speculate that in the arterial approach group, diabetes may affect AVF maturation and patency by aggravating endothelial dysfunction and oxidative stress, whereas in the venous approach group, the inflammatory response induced by repeated puncture injuries may increase the effects of high FDP and D-Dimer on vascular remodeling and thrombosis. Notably, this study revealed for the first time that FDP levels have a U-shaped relationship with AVF patency, suggesting that FDP may have a biphasic effect on vascular remodeling. We speculate that a slight increase in FDP may reflect the synthesis and metabolism of fibrin during vascular remodeling, whereas excessive elevation suggests excessive fibrin deposition and an increased risk of thrombosis. Our findings offer valuable insights that can potentially enhance postoperative care, aligning with the principles of evidence-based practice in optimizing patient outcomes following surgery. However, the exact mechanism is still unclear and requires further research to clarify the role of FDP in AVF stenosis and to verify the optimal FDP level range. Although a U-shaped association was observed between FDP levels and arteriovenous fistula patency, its validity requires further verification through external validation.

The limitations of this study include its retrospective, single-center design and possible selection bias. Due to the inherent limitation of the retrospective database, data regarding baseline medication use (including antiplatelet and anticoagulant agents), history of ipsilateral central venous catheterization, and vascular patency rates were completely unavailable. Because the choice of access route was operator-dependent, the anatomical characteristics of the lesion likely influenced both the selection of the approach and the treatment outcomes. This introduces selection bias, which represents a major limitation of this study. The sample size and the time frame of our study are also potential limitations that could affect the universal applicability of our findings. Future studies with larger sample sizes are needed to confirm our findings, which should also be further confirmed through external cohort studies or prospective multicentre study. Although the results support the superiority of the arterial approach to the PTA, the approach should be flexibly selected based on the patient’s condition in clinical practice. For patients who cannot be successfully punctured through the artery or have a high risk of arterial stenosis, the venous approach is still a necessary choice. Future studies should expand the spectrum of risk factors to include markers of vascular aging, calcification, inflammation, coagulation, and endothelial function to establish a more complete AVF stenosis risk prediction model to guide individualized monitoring and intervention.

In summary, this study provides strong evidence supporting the superiority of the arterial approach in PTA over the venous approach in treating stenosis near AVF anastomosis. We also found that diabetes, low blood flow, and elevated FDP and D-Dimer are risk factors for AVF stenosis, and the relevant factors differ between different approach methods. These results can guide approach-based individualized risk assessment and management strategies to optimize AVF interventional treatment and guide individualized and precise vascular access monitoring and intervention, and ultimately improving the prognosis of hemodialysis patients.

## Data Availability

The original contributions presented in the study are included in the article/Supplementary material, further inquiries can be directed to the corresponding authors.
