# Supplementary material for: Efficacy study of juxta-anastomotic stenosis of the arteriovenous fistula via percutaneous transluminal angioplasty with different accesses to the arteries and veins
Source: Front Med (Lausanne). 2026 Jul 22;13:1883032. doi: 10.3389/fmed.2026.1883032 (PMC13439415; doi:10.3389/fmed.2026.1883032)
Supplement: Supplementary file 1 [file Table_1.DOCX]

**Supplementary Material**

| **Table S1. Univariate and multivariate Cox regression analyses affecting 6 months post-PTA in the artery group** | | | | |
| --- | --- | --- | --- | --- |
|  | Univariable analysis | | Multivariable analysis | |
| Predictors | HR (95% CI) | *P-*value | HR (95% CI) | *P-*value |
| Sex | 0.74 (0.39,1.41) | 0.363 |  |  |
| Age | 1.00 (0.98,1.03) | 0.804 |  |  |
| BMI | 1.00 (0.91,1.11) | 0.938 |  |  |
| Hypertension | 0.99 (0.45,2.16) | 0.983 |  |  |
| Diabetes | 2.17 (1.13,4.17) | 0.018 | 2.28 (1.14~4.53) | 0.019 |
| Carcinoma | 1.35 (0.42,4.40) | 0.631 |  |  |
| Peripheral vascular disease | 0.89 (0.46,1.72) | 0.724 |  |  |
| Hyperlipidemia | 1.45 (0.52,4.10) | 0.500 |  |  |
| Preoperative blood flow | 0.99 (0.99,1.00) | 0.166 | 1.00(0.99,1.00) | 0.113 |
| WBC | 1.01 (0.89,1.13) | 0.908 |  |  |
| Neutrophil ratio | 0.35 (0.02,7.81) | 0.512 |  |  |
| FDP | 1.07 (0.98,1.15) | 0.145 | 1.06(0.97,1.16) | 0.167 |
| D-Dimer | 1.07 (0.98,1.17) | 0.208 |  |  |
| Creatinine | 0.99 (0.99,1.00) | 0.796 |  |  |

| **Table S2. Univariate and multivariate Cox regression analyses affecting 6 months post-PTA in the vein group** | | | | |
| --- | --- | --- | --- | --- |
|  | Univariable analysis | | Multivariable analysis | |
| Predictors | HR (95% CI) | *P-*value | HR (95% CI) | *P-*value |
| Sex | 0.81 (0.49,1.35) | 0.424 |  |  |
| Age | 1.01 (0.99,1.03) | 0.19 | 1.01(0.99~1.03) | 0.619 |
| BMI | 1.02 (0.96,1.09) | 0.498 |  |  |
| Hypertension | 0.67 (0.35,1.29) | 0.253 |  |  |
| Diabetes | 1.60 (0.97,2.66) | 0.069 | 1.43(0.82~2.49) | 0.202 |
| Carcinoma | 4.85 (1.51,15.59) | 0.008 |  |  |
| Peripheral vascular disease | 0.61 (0.15,2.48) | 0.451 |  |  |
| Hyperlipidemia | 0.81 (0.48,1.34) | 0.401 |  |  |
| Preoperative blood flow | 0.99 (0.99,1.00) | 0.419 |  |  |
| WBC | 0.95 (0.86,1.05) | 0.305 |  |  |
| Neutrophil ratio | 0.24 (0.02,2.42) | 0.235 |  |  |
| FDP | 1.11 (1.03,1.21) | 0.012 | 1.10 (1.01~1.19) | 0.029 |
| D-Dimer | 1.09 (1.02,1.17) | 0.022 | 1.09 (1.02~1.17) | 0.014 |
| Creatinine | 0.99 (0.99,1.00) | 0.683 |  |  |

| **Table S3. Complications in the artery and vein groups** | | | | |
| --- | --- | --- | --- | --- |
|  | Complication  Multivariable analysis | | | |
| Group | Thrombus | Occlusion | Pseudoaneurysm | Aneurysmal dilatation |
| Artery | 16（55%） | 9（31%） | 2（7%） | 2（7%） |
| Vein | 32（59%） | 20（37%） | 1（2%） | 1（2%） |
| χ^2^ | 0.299 | 0.129 | 0.311 | 0.311 |
| *P*-value | 0.584 | 0.719 | 0.577 | 0.577 |

Figure S1. A Kaplan‒Meier curve was used to verify the risk factors and protective factors that affect the rate of AVF stenosis after interventional treatment in the arterial (Fig A) and vein (Fig B,C) group, and the related factors included diabetes (Fig A), D-Dimer (Fig B) and FDP（Fig C）.
